# Reverse Potts for the Treatment of Severe Idiopathic Pulmonary Hypertension in Children

**DOI:** 10.21470/1678-9741-2022-0320

**Published:** 2023-06-14

**Authors:** Marcelo Frederigue de Castro, Edmundo Clarindo Oliveira, Maria Carmo Pereira Nunes, Carla de Oliveira, Maria Gabriela Costa de Almeida, Jose Augusto Almeida Barbosa

**Affiliations:** 1 Department of Internal Medicine, Hospital Vila da Serra, Nova Lima, Minas Gerais, Brazil; 2 Department of Internal Medicine, Universidade Federal de Minas Gerais, Belo Horizonte, Minas Gerais, Brazil; 3 Department of Cardiac Surgery, Hospital Vila da Serra, Nova Lima, Minas Gerais, Brazil; 4 Department of Pediatric Cardiology, Hospital Vila da Serra, Nova Lima, Minas Gerais, Brazil; 5 Department of Pediatric Cardiology, Hospital Felicio Rocho, Belo Horizonte, Minas Gerais, Brazil

**Keywords:** Pulmonary Vascular Resistance, Hypertension, Reverse Potts, Palliative treatment

## Abstract

Idiopathic pulmonary arterial hypertension is a rare and progressive disease with poor prognosis. Many patients progressively worsen even when using combinations of specific drugs for its treatment. Herein, we present our experience in the management of three children with severe pulmonary arterial hypertension refractory to clinical treatment who underwent Potts surgery in addition to clinical treatment.

**Table:** 

Abbreviations, Acronyms & Symbols	
CHF	= Congestive heart failure
PA	= Pulmonary artery
PAH	= Pulmonary arterial hypertension
PH	= Pulmonary hypertension
PP	= Pulmonary pressure
PTFE	= Polytetrafluoroethylene
RV	= Right ventricle
SpO₂	= Oxygen saturation
WHO	= World Health Organization

## INTRODUCTION

Pulmonary arterial hypertension (PAH), in the absence of a removable cause, is a progressive disease with poor prognosis^[1]^. There is no ideal medication, and even with several specific drugs for the treatment of PAH patients, many of them progress with worsening and are refractory to double or triple treatment, thereby requiring other measures. Lung transplantation is reserved for such patients at the advanced stage of the disease^[1,2]^.

Other palliative options, such as atrial septostomy, allow the balance of pressure between the atria and the reduction in systemic venous hypertension with improvement of congestive heart failure (CHF); however, it presents the risk of severe hypoxemia with progression to death and is generally not recommended when the right atrial pressure is > 20 mmHg^[2-5]^. On the other hand, Potts surgery (connection between the left pulmonary artery [PA] and the descending aorta) was performed to allow a shunt between the PA and aorta and avoid a suprasystemic increase in pulmonary pressure (PP) and the consequent relief of the right ventricle (RV) with promising results in selected cases. In this situation, as the intention is to allow shunting from right to left when necessary, it is called reverse Potts^[6]^.

## CASE PRESENTATION

Case 1. A male patient was diagnosed at six years of age with idiopathic PAH without response to the pulmonary vasoreactivity test with nitric oxide at a pulmonary/systemic pressure ratio of 0.78. At that time, treatment with specific vasodilators was initiated. Four years later, the patient returned to World Health Organization (WHO) functional class IV with severe CHF, cough, and syncope upon slight exertion. The echocardiogram showed large right ventricular dilation with severe dysfunction, pericardial effusion, and left ventricular septal deviation. In addition to treatment with sildenafil, bosentan, and the treatment of CHF, prostacyclin was administered, without response. After discussion with the family, the intensive care unit and pediatrics team was referred to perform a reverse shunt. Initially, in a hybrid room, cardiac catheterization was performed with aortography without finding any aortopulmonary communication that could be amplified by catheterization. The pulmonary/systemic pressure ratio was 1.35. Shunting was performed with a 7-mm diameter tube implant without complications. The patient progressed with a slight improvement allowing for extubation, but the echocardiogram showed a dysfunction of the RV already in an irreversible phase even after the therapeutic measures and performing the Potts procedure. The patient presented with severe hemoptysis after a peak of pulmonary hypertension followed by death 15 days after surgery.

Case 2. A male patient was diagnosed with severe primary pulmonary hypertension (PH) with systemic PP at six months of age and without response to the vasoreactivity test with nitric oxide. Treatment was initiated with sildenafil, and then combined with bosentan, with slight improvement. The patient progressed with worsening of symptoms, presenting several episodes of syncope every day. The echocardiogram showed suprasystemic PP at rest and good biventricular function. After a multidisciplinary meeting and parental consent, Potts surgery was performed at three years of age with a 6-mm diameter tube implant. The patient showed good progress in the immediate postoperative period. During follow-up, there was a decrease in saturation to 84% in the lower limbs and to 95% in the upper limbs, with abdominal pain and a slight increase in liver enzymes that persisted for three months, requiring hospitalization with symptomatic treatment. The patient showed progressive improvement, without syncope and with normal physical activity for his age. Currently, the patient is undergoing three years of follow-up with quarterly regular clinical control, WHO functional class II, systemic PP, and has shown good biventricular function with oxygen saturation (SpO₂) in the upper limb and lower limb of 97% and 89%, respectively.

Case 3. This is a female patient, with 2.8 kg birth weight, persistent tachypnea, and severe PH that was maintained after three months, with no identified cause after a full investigation. She was treated initially with sildenafil, and then sildenafil combined with bosentan, a diuretic, without significant improvement. The echocardiogram showed systemic PP at rest and suprasystemic PP with crying. After a multidisciplinary discussion of the case and with parental consent, she was submitted to Potts surgery at 10 months of age with interposition of a 6-mm tube. Treatment with sildenafil and bosentan was maintained, with improvement, allowing extubation and maintenance in room air. She remained stable during the ten-month follow-up with SpO₂ > 94% in the upper and lower limbs and a pulmonary/systemic pressure ratio of 0.80.

**Fig. 1:**
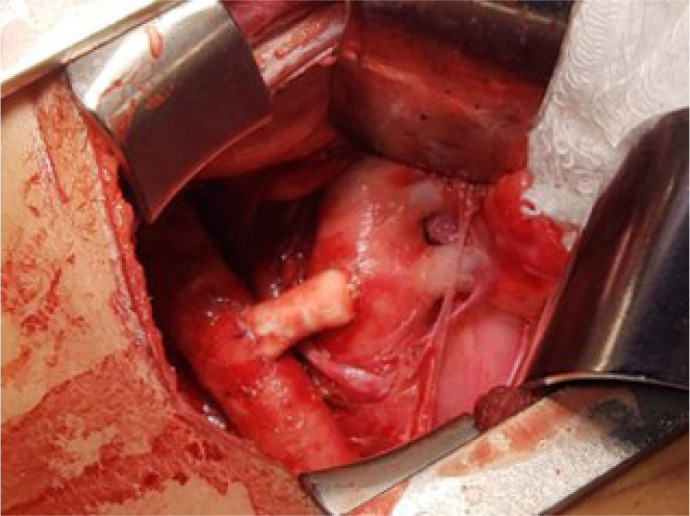
End-to-side anastomosis; polytetrafluoroethylene prosthesis between the descending thoracic aorta and the left branch of the pulmonary artery.

Surgical access is obtained by left posterolateral thoracotomy at the level of the fourth intercostal space, with intrapleural dissection of the descending thoracic aorta, in the segment between the origin of the left subclavian artery and the middle third of the descending thoracic aorta, as well as the left pulmonary branch. Next, systemic heparinization (100 U/kg heparin) is performed. Partial clamps are applied to the medial face of the thoracic aorta and the right PA. Subsequently, longitudinal arteriotomies are performed, and end-to-side anastomoses are formed between the PA and the descending aorta with interposition of polytetrafluoroethylene (PTFE) prosthesis of the chosen diameter, with 7.0 continuous PROLENE® sutures. The reversal of heparinization with protamine sulfate is performed only if there is abundant diffuse bleeding through the sutures. The choice of PTFE prosthesis size is made according to the relationship between the vessels and their respective diameters. They are usually between 6 and 8 mm in diameter. Care must be taken to avoid distortions and kinking, and the preference in positioning it is the least angled possible and with the shortest possible length. Figure 1 shows the final result of the surgery.

## DISCUSSION

The specific treatment of PH aims to control PP and improve cardiac output, with a consequent increase in patients’ longevity and quality of life. Knowledge that patients with Eisenmenger syndrome have greater longevity and better functional class than patients with PAH^[6,7]^ with the same PP level, aroused interest in creating a left-to-right shunt in this special group of patients, aiming to improve their functional class, prolong survival, and even to await the possibility of lung transplantation. Provisions to allow right-to-left atrial or arterial (pulmonary-aortic) shunting to improve the performance of the RV have been used. The creation of anastomosis between the left branch of the PA and the descending aorta to relieve PH with a right-to-left shunt (Potts surgery with reverse shunt) has the benefits of preventing the suprasystemic increase in PP, mitigating the consequences in the RV and avoiding low oxygenation of the coronaries and brain^[7,8]^. Among the three cases presented, there was improvement in two cases, as mentioned above. The questions that remain unanswered are as follows: What is the ideal time to indicate the creation of a shunt? And should it be performed only in patients with suprasystemic pressure? We believe that the best time would be when refractoriness to PP treatment is observed before irreversible right ventricular dysfunction occurs. Teamwork that regularly analyzes the clinical and laboratory parameters of patients is essential to choose the ideal time and achieve better results.

## CONCLUSION

Palliative treatment using reverse Potts surgery in patients with idiopathic pulmonary hypertension, when well indicated, is an alternative to prolong and improve the quality of life of these patients. The decision whether or not to perform the procedure must be made by a multidisciplinary team.

**Table:** 

Authors’ Roles & Responsibilities	
MFC	Substantial contributions to the conception or design of the work; or the acquisition, analysis, or interpretation of data for the work; drafting the work or revising it critically for important intellectual content; final approval of the version to be published
ECO	Substantial contributions to the conception or design of the work; or the acquisition, analysis, or interpretation of data for the work; drafting the work or revising it critically for important intellectual content; final approval of the version to be published
MCPN	Substantial contributions to the conception or design of the work; or the acquisition, analysis, or interpretation of data for the work; drafting the work or revising it critically for important intellectual content; final approval of the version to be published
CO	Substantial contributions to the conception or design of the work; or the acquisition, analysis, or interpretation of data for the work; drafting the work or revising it critically for important intellectual content; final approval of the version to be published
MGCA	Substantial contributions to the conception or design of the work; or the acquisition, analysis, or interpretation of data for the work; drafting the work or revising it critically for important intellectual content; final approval of the version to be published
JAAB	Substantial contributions to the conception or design of the work; or the acquisition, analysis, or interpretation of data for the work; drafting the work or revising it critically for important intellectual content; final approval of the version to be published
